# Optimizing human hepatocyte models for metabolic phenotype and function: effects of treatment with dimethyl sulfoxide (DMSO)

**DOI:** 10.14814/phy2.12944

**Published:** 2016-11-02

**Authors:** Nikolaos Nikolaou, Charlotte J. Green, Pippa J. Gunn, Leanne Hodson, Jeremy W. Tomlinson

**Affiliations:** ^1^ Oxford Centre for Diabetes Endocrinology & Metabolism and NIHR Biomedical Research Centre University of Oxford Churchill Hospital, Headington Oxford U.K.

**Keywords:** Carbohydrate, DMSO, HepG2, lipid, metabolism

## Abstract

Primary human hepatocytes are considered to be the “gold standard” cellular model for studying hepatic fatty acid and glucose metabolism; however, they come with limitations. Although the HepG2 cell line retains many of the primary hepatocyte metabolic functions they have a malignant origin and low rates of triglyceride secretion. The aim of this study was to investigate whether dimethyl sulfoxide supplementation in the media of HepG2 cells would enhance metabolic functionality leading to the development of an improved in vitro cell model that closely recapitulates primary human hepatocyte metabolism. HepG2 cells were cultured in media containing 1% dimethyl sulfoxide for 2, 4, 7, 14, and 21 days. Gene expression, protein levels, intracellular triglyceride, and media concentrations of triglyceride, urea, and 3‐hydroxybutyrate concentrations were measured. Dimethyl sulfoxide treatment altered the expression of genes involved in lipid (FAS, ACC1, ACC2, DGAT1, DGAT2, SCD) and glucose (PEPCK, G6Pase) metabolism as well as liver functionality (albumin, alpha‐1‐antitrypsin, AFP). mRNA changes were paralleled by alterations at the protein level. DMSO treatment decreased intracellular triglyceride content and lactate production and increased triglyceride and 3‐hydroxybutyrate concentrations in the media in a time‐dependent manner. We have demonstrated that the addition of 1% dimethyl sulfoxide to culture media changes the metabolic phenotype of HepG2 cells toward a more primary human hepatocyte phenotype. This will enhance the currently available in vitro model systems for the study of hepatocyte biology related to pathological processes that contribute to disease and their response to specific therapeutic interventions.

## Introduction

The liver is the major metabolic visceral organ in the human body and performs over 500 different functions (Naruse et al. 2007) including bile and albumin production and secretion, foreign and metabolic poison detoxification, and metabolism of lipids, carbohydrates, and vitamins (Sherlock 1978). Diseases of the liver are increasing in prevalence; for example, nonalcoholic fatty liver disease (NAFLD), which encompasses a spectrum ranging from simple steatosis to fibrosis, cirrhosis, and hepatocellular carcinoma (Trauner et al. 2010) is now one of the most common liver diseases in Western countries (de Alwis and Day 2008). Investigating the mechanisms that underpin the development and progression of NAFLD, as well as potential treatments, requires in vitro cellular models that mimic human hepatocyte metabolism and functionality.

The liver comprised parenchymal (hepatocytes) and nonparenchymal cells (including Kupffer cells, hepatic stellate cells), with hepatocytes contributing approximately 80% of cellular liver mass. Primary human hepatocytes are therefore the ideal in vitro cellular model to investigate aspects of liver function and metabolism as they most closely recapitulate the in vivo liver (Green et al. 2015). Although human primary hepatocytes are considered the gold standard, there are significant challenges in their use, such as availability of cells, donor variability, inability to proliferate, and a short life span (Green et al. 2015). Therefore, a variety of human hepatoma cell lines have been generated to investigate human hepatocyte metabolism and function, including the HepG2, Hep3b, and Huh7.0 cell lines. The HepG2 cell line has been extensively used in cell‐based metabolic studies; however, because of their malignant origin, they tend to present with an abnormal metabolic phenotype compared to human primary cells. As an example, HepG2 cells have increased rates of de novo lipogenesis and glycolysis (Pereira da Silva et al. 2009; Daniels et al. 2014).

The amphipathic molecule dimethyl sulfoxide (DMSO) has multiple effects on cellular function (Santos et al. 2003; Pal et al. 2012). A number of previous studies have examined whether DMSO treatment alters hepatoma cell line functionality. However, no study published to date has examined in detail the impact of DMSO treatment of carbohydrate and lipid metabolism. DMSO treatment of HepG2 and Huh7.0 cells has been reported to enhance drug metabolism (Choi et al. 2009) and reduce lipid accumulation (Song et al. 2012), although the mechanisms underpinning these observations remain to be elucidated. Moreover, it remains unclear whether DMSO treatment of hepatoma cell lines affects carbohydrate metabolism. The aim of this study was therefore to determine whether DMSO treatment could enhance HepG2 cell metabolic functionality leading to the development of a novel and improved in vitro model that may more closely resembles primary human hepatocytes.

## Methods and Materials

### C3A and HepG2 cell culture and treatments

HepG2 cells were a gift from Dr. Karl Morten (University of Oxford) and were cultured in Dulbecco's minimum essential medium (DMEM) (Zen Bio Inc., Durham, NC), containing 4.5 g/L glucose, and supplemented with 10% fetal bovine serum, 1% penicillin/streptomycin, and 1% non‐essential amino acids. The C3A human hepatocyte cell line was purchased from LGC Standards (ATCC – CRL‐10741, Middlesex, UK), and cultured in Eagle's minimum essential medium (EMEM) (Sigma‐Aldrich, Dorset, UK) containing 4.5 g/L glucose, and supplemented with 10% fetal bovine serum, 1% penicillin/streptomycin, and 1% nonessential amino acids. For all treatments, cells were seeded in six‐well Cell Bind plates (Appleton Woods, Birmingham, UK) at a cell density of 3.5 × 10^5^ cells per well. At 90% confluence, culture medium was replaced with 2 mL complete medium containing 1% DMSO (Sigma‐Aldrich, Dorset, UK). Based on a previously described protocol (Sainz and Chisari 2006), cultures were incubated for 2, 4, 7, 14, and 21 days, during which time complete DMSO‐containing medium was replenished every 2 days. Non‐DMSO‐treated cells were also seeded in six‐well Cell Bind plates at the same cell density until 90% confluence (approximately 5 days), then medium was replenished for 2 days and samples were collected. An important methodological consideration is that it is not possible to have control cells that were “time matched” in non‐DMSO‐supplemented media. Both HepG2 and C3A cells will continue to proliferate and it is well established that there is increased spontaneous cell loss in the postconfluent stage (Hosick 1976). We observed increased cell death in HepG2 cells after 9–10 days in cell culture without passage. For biochemical measurements using media, cells were cultured in complete media without phenol red +/− DMSO for 2, 4, 7, 14, and 21 days.

### Primary human hepatocytes

Liver was obtained from patients undergoing surgery who had consented (NRES Committee South Central; Berkshire B 11/SC/0443) to the use of excess tissue (resection surplus) for research. Hepatocytes were dissociated from whole liver tissue via a two‐stage collagenase digestion as described previously by Green et al. (2015). Cells were diluted to 1 million cells per mL in William's E buffer containing hepatocyte supplements before plating on Type I collagen coated plates. Cells were collected 24 h postseeding for RNA isolation.

### Fatty acid treatments

In media lacking phenol red, cells were treated with 0.25% fatty acid‐free BSA alone or conjugated to 45 μmol/L oleic, 30 μmol/L palmitic, and 25 μmol/L linoleic acid (OPL; physiological ratio 45:30:25%) for 24 or 48 h (Green et al. 2015).

### RNA extraction and reverse transcription

Total RNA was extracted from cells using the Tri‐Reagent system (Sigma‐Aldrich, Dorset, UK). Concentration was determined spectrophotometrically at OD260 on a Nanodrop spectrophotometer (Thermo Scientific, Hemel Hempstead, UK). In a 20‐μL volume, 1 μg of total RNA was incubated with 10× RT buffer, 100 mmol/L dNTP mix, 10× RT random primers, 50 U/μL MultiScribe reverse transcriptase, and 20 U/μL RNase inhibitor. The reverse transcription was carried out at 25°C for 10 min, 37°C for 120 min, and then the reaction was terminated by heating to 85°C for 5 min.

### Real‐time PCR

All real‐time PCR experiments were conducted using an ABI 7900HT sequence detection system (Perkin‐Elmer Applied Biosystems, Warrington, UK). Reactions were performed in 6 μL volumes on 384‐well plates in reaction buffer containing 3 μL of 2× KlearKall Master Mix with standard ROX (LGC Genomics, Middlesex, UK). Life Technologies supplied all primers and reactions normalized against the housekeeping gene GAPDH, provided as a preoptimized control probe. All target genes were labeled with FAM. The reaction conditions were as follows: 95°C for 10 min, then 40 cycles of 95°C for 15 sec, and 60°C for 1 min. The Ct (dCt) of each sample using the following calculation (where E is reaction efficiency – determined from a standard curve): ΔCt = E^[min Ct−sample Ct]^ (Pfaffl 2001), using the 1/40 dilution from a standard curve generated from a pool of all cDNAs as the calibrator for all samples. The relative expression ratio was calculated using the following: Ratio = ΔCt_[target]_/ΔCt_[ref]_ (Pfaffl 2001) and expression values were normalized to 18 sec.

### Protein extraction and immunoblotting

Total protein was extracted from cells using RIPA buffer (150 mmol/L NaCl, 1.0% IGEPAL^®^ CA‐630, 0.5% sodium deoxycholate, 0.1% SDS, and 50 mmol/L Tris, pH 8.0) (Sigma‐Aldrich, Dorset, UK), and protease inhibitor cocktail (ThermoFisher Scientific, Loughborough, UK). Protein concentrations were measured using a commercially available assay (Bio‐Rad Laboratories Inc., Hercules, CA). Protein levels were determined using the Bio‐Rad assay kit, according to the manufacturer's protocol (Bio‐Rad, Hemel Hempstead, UK). Primary (G6PC [Abcam plc, Cambridge, UK], PEPCK, IRE1, CHOP, *β*‐tubulin [Cell Signalling, Danvers]) and secondary antibodies from Dako were used at a dilution of 1/1000 and 1/2000, respectively. To ascertain equal gel loading, proteins were normalized to *β*‐tubulin. Bands were visualized with ECL (Pierce Thermo Fisher Scientific) and ChemiDocXS imager (Bio‐Rad). Western blots were quantified by densitometry analysis using ImageJ (NIH, Bethesda, MD, http://rsb.info.nih.gov/ij).

### Oil red O staining

Cells were washed twice in PBS and fixed using 10% formalin for 1 h. Cells were then washed in 60% isopropanol and left to dry. Staining was carried out by adding Oil red O (6 mmol/L) (Sigma‐Aldrich, Dorset, UK) for 15 min at room temperature. Cells were then counterstained using hematoxylin and eosin and mounted onto slides using aqua mount.

### Urea, triglyceride (TG), and 3‐hydroxybutyrate (3OHB) concentrations

Urea and TG (intracellular and media) and 3OHB were analyzed using Instrumentation Laboratory kits on an ILab 650 Clinical Chemistry analyzer as described previously (Green et al. 2015; McNeil et al. 2014). Complete medium‐containing FBS or lysis buffer were used as background controls.

### Statistical analysis

For multiple comparisons, statistical analysis was performed using ordinary one‐way analysis of variance (ANOVA) and control versus different time points were compared. Statistical analysis on qPCR data was performed on mean relative expression ratio values (Ratio = ΔCt_[target]_/ΔCt_[ref]_) (Pfaffl 2001). Data analysis was performed using GraphPad Prism software (GraphPad Software Inc., La Jolla) and considered statistically significant at *P* < 0.05.

## Results

### DMSO treatment of human hepatoma cell lines alters the expression of liver function markers

**Figure 1:**
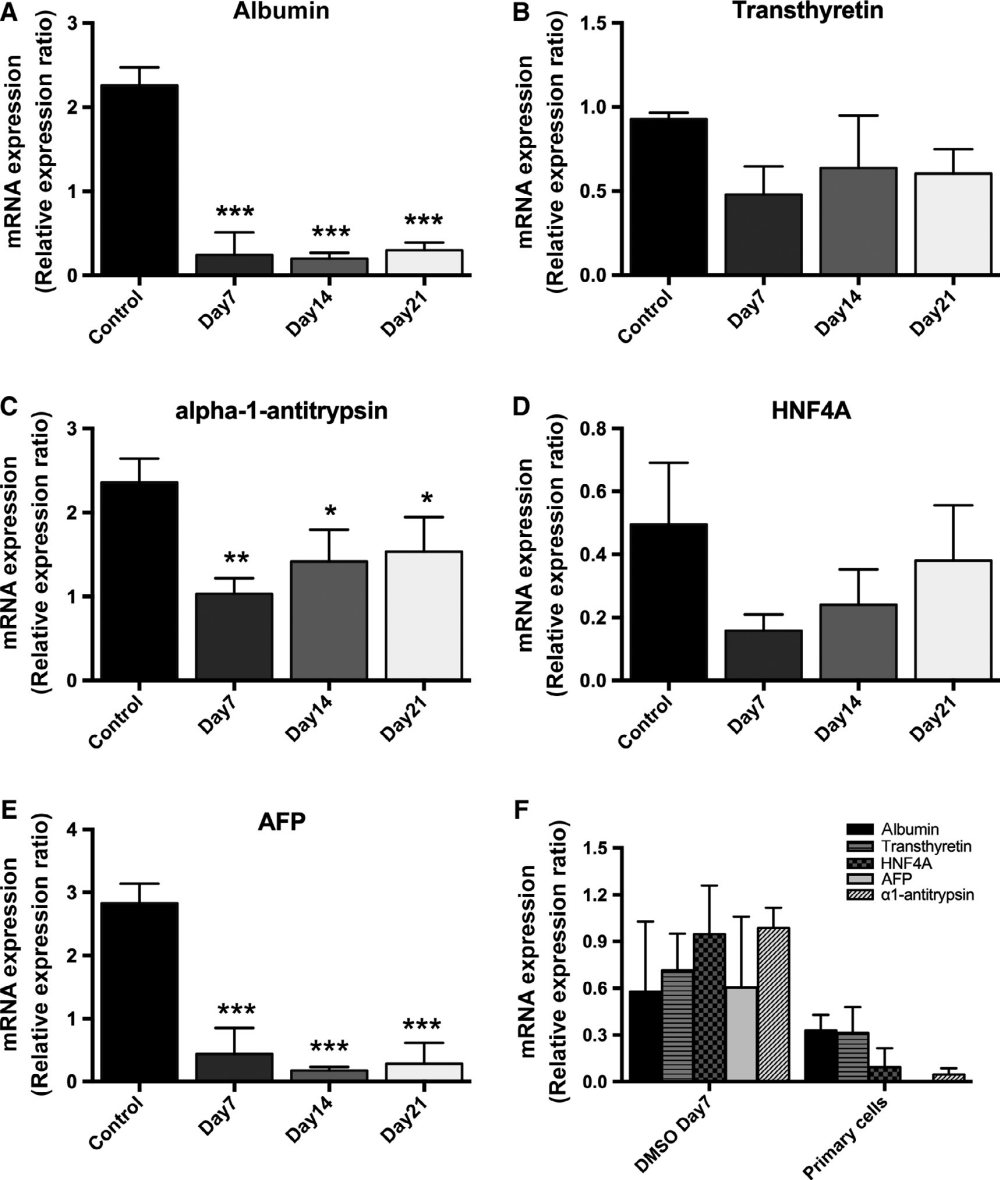
DMSO treatment causes a time‐dependent change in mRNA expression of liver function markers albumin, transthyretin, alpha‐1‐antitrypsin, HNF4A, and AFP in HepG2 cells (A–E). Furthermore, DMSO treatment of HepG2 cells for 7 days reduces the mRNA expression of these markers to levels that are similar to those seen in primary human hepatocytes (F). Data presented are mean ± SD of *n* = 3 experiments performed in triplicate, *P < 0.05, **P < 0.01, ***P < 0.001, control versus day of DMSO treatment. The relative expression ratio was calculated using the following: Ratio = ΔCt_[target]_/ΔCt_[ref]_ (Pfaffl 2001) and expression values were normalized to 18 sec.

Expression levels of four hepatic function markers (albumin, HNF4A, transthyretin, and alpha‐1‐antitrypsin) were measured in confluent untreated and DMSO‐treated HepG2 and C3A cells. mRNA expression levels of many genes were higher in the untreated compared to DMSO‐treated HepG2 cells (Fig. 1A–D). Importantly, mRNA expression of the tumor marker alpha‐fetoprotein (AFP) decreased after DMSO treatment, with a significant decrease observed after 7 days that persisted up to 21 days of treatment (Fig. 1E). Urea secretion, a measure of hepatic cell function, was not compromised by DMSO treatment in HepG2 cells (0.037 ± 0.01 [control] vs. 0.023 ± 0.007 [day 7], 0.039 ± 0.01 [day 14], 0.036 ± 0.02 μmol/mg protein [day 21]). To further confirm the effect of DMSO on human hepatoma cell lines, C3A cells were also used as an additional cellular model. The C3A cell line is a derivative of the HepG2 cell line and has also used for a number of metabolic studies (Iyer et al. 2010; Nasiri et al. 2015). Similar observations were made in C3A cells treated with DMSO and absolute changes in mRNA expression levels are presented in Table 1.

**Table 1:** The impact of DMSO to modulate gene expression as measured by qPCR in human hepatoma C3A cells

C3A	Control	Day 7	Day 14	Day 21
Albumin	3.59 ± 0.66	1.04 ± 0.17***	0.42 ± 0.03***	0.77 ± 0.1***
*α*1‐Antitrypsin	2.18 ± 0.25	1.29 ± 0.13***	1.51 ± 0.12**	2.19 ± 0.21
Transthyretin	2.86 ± 0.49	1.39 ± 0.13***	0.93 ± 0.1***	1.31 ± 0.26***
HNF4A	0.98 ± 0.25	0.45 ± 0.12*	0.44 ± 0.12*	0.8 ± 0.24
AFP	4.08 ± 0.17	1.58 ± 0.08***	0.33 ± 0.04***	0.51 ± 0.1***
FAS	3.98 ± 0.73	1.63 ± 0.45**	1.76 ± 0.17**	2.7 ± 0.78
ACC1	5.44 ± 1.37	2.06 ± 0.43**	1.31 ± 0.17***	2.08 ± 0.27**
ACC2	2.6 ± 0.43	1.82 ± 0.46*	1.54 ± 0.09**	2.21 ± 0.08
SCD	2.04 ± 0.34	1.75 ± 0.3	1.01 ± 0.09*	2.04 ± 0.45
DGAT1	4.77 ± 0.5	1.8 ± 0.32***	1.66 ± 0.27***	2.14 ± 0.06***
DGAT2	3.05 ± 0.56	0.82 ± 0.15***	0.47 ± 0.04***	0.88 ± 0.27***
PEPCK	0.01 ± 0.002	0.19 ± 0.03*	0.31 ± 0.04**	0.88 ± 0.14***
G6PC	0.03 ± 0.01	0.03 ± 0.006	0.08 ± 0.009**	0.12 ± 0.03***

Data are presented as mean ± SD of relative expression ratio, *P < 0.05, **P < 0.01, ***P < 0.001. The relative expression ratio was calculated using the following: Ratio = ΔCt_[target]_/ΔCt_[ref]_ (Pfaffl 2001) and expression values were normalized to 18 sec.

In order to ascertain if DMSO treatment of HepG2 cells induced a phenotype more reflective of primary human hepatocytes, the mRNA expression profile of liver function markers at day 7 of DMSO treatment was compared with that of primary cultures of human hepatocytes. At day 7, DMSO‐treated cells had reached 100% confluence and demonstrated decreased proliferation rate. DMSO treatment (7 days) reduced mRNA levels of liver function makers to levels similar to those seen in primary human hepatocytes (Fig. 1F).

### DMSO treatment of HepG2 cells decreases the expression of ER stress markers

**Figure 2:**
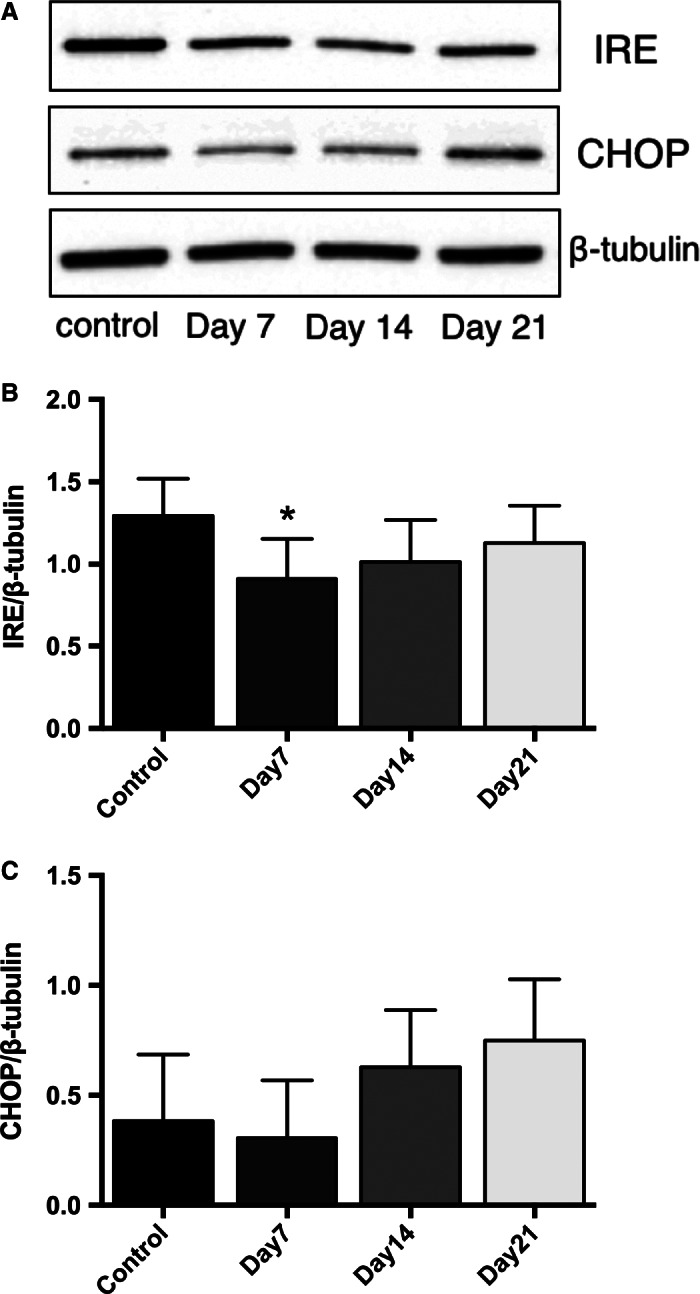
DMSO treatment decreases the expression of the ER stress markers IRE and CHOP in HepG2 cells, as shown by western blotting experiments. (A). Protein expression levels of IRE and CHOP were normalized against *β*‐tubulin (B–C). Data presented are mean ± SD of *n* = 3–5 experiments, *P < 0.05, control versus day of DMSO treatment.

ER stress has the potential to influence hepatocyte functionality and metabolism and therefore markers of convergent ER stress pathways were measured across the time course of the experimental protocol. IRE‐1 protein expression decreased after 7 days of DMSO treatment, while there was no significant change in CHOP protein levels (Fig. 2A–C).

### DMSO treatment changes the lipid metabolic phenotype of HepG2 cells

**Figure 3:**
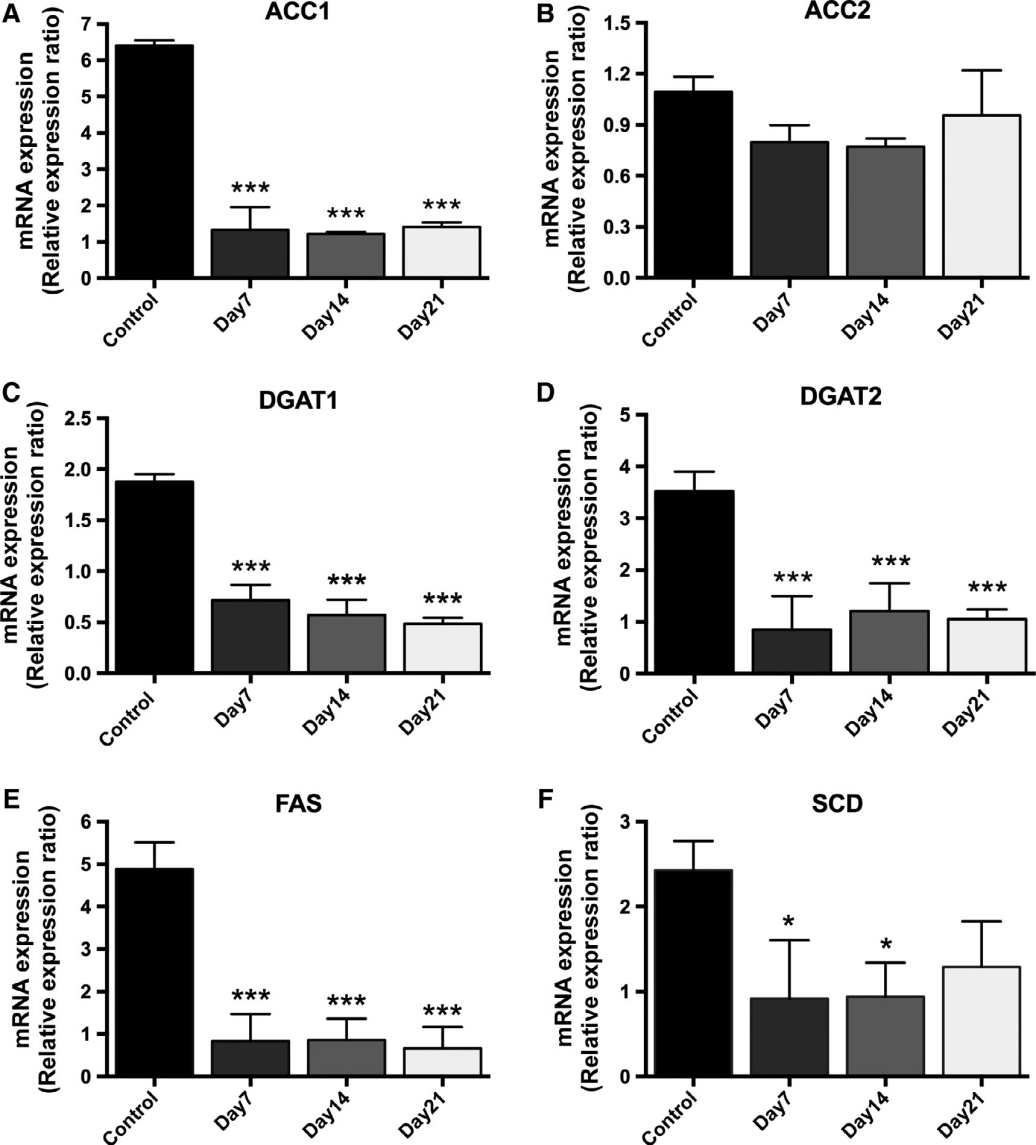
DMSO treatment decreases the mRNA expresion of the fatty acid synthetic and oxidation genes acetyl‐CoA carboxylase alpha (ACC1), acetyl‐CoA carboxylase beta (ACC2), diacylglycerol O‐acyltransferase 1 (DGAT1), diacylglycerol O‐acyltransferase 2 (DGAT2), fatty acid synthase (FAS), and stearoyl‐CoA desaturase (SCD) in a time‐dependent manner in HepG2 cells. Data presented are mean ± SD of *n* = 3 experiments performed in triplicate, *P < 0.05, **P < 0.01, ***P < 0.001, control versus day of DMSO treatment. The relative expression ratio was calculated using the following: Ratio = ΔCt_[target]_/ΔCt_[ref]_ (Pfaffl 2001) and expression values were normalized to 18 sec.

The fatty acid synthetic and oxidation genes, acetyl‐CoA carboxylase alpha (ACC1), acetyl‐CoA carboxylase beta (ACC2), diacylglycerol O‐acyltransferase 1 (DGAT1), diacylglycerol O‐acyltransferase 2 (DGAT2), fatty acid synthase (FAS), and stearoyl‐coA desaturase (SCD) mRNA expression were significantly decreased in HepG2 cells after DMSO treatment in a time‐dependent manner (Fig. 3A–F). Observations were similar in C3A human hepatoma cells (Table 1).

**Figure 4:**
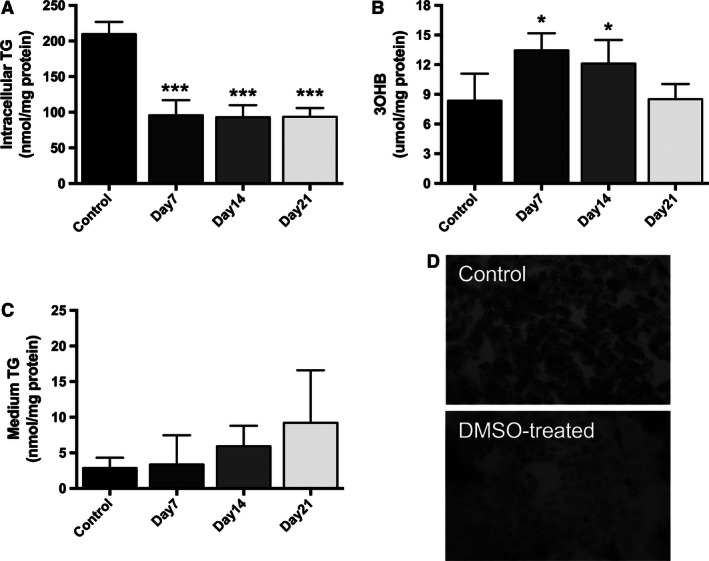
DMSO treatment reduces the intracellular TG content in a time‐dependent manner in HepG2 cells (A). These observations were mirrored by a decrease in intracellular neutral lipid (TG) staining with oil red O, after 7 days of DMSO treatment (D). Furthermore, 3OHB levels are significantly increased in HepG2 cell medium after treatment with 1% DMSO for 7 days (B). In addition, DMSO treatment appears to increase the TG concentration in the medium from HepG2 cells in at time‐dependent manner (C). Data presented are mean ± SD of *n* = 3–5 experiments, *P < 0.05, ***P < 0.001, control versus day of DMSO treatment.

Fatty acids within the hepatocyte can be partitioned into esterification (to synthesize TG) or oxidation (resulting in the generation of 3OHB) pathways. Compared with untreated control cells, DMSO‐treated HepG2 cells had significantly lower concentrations of intracellular TG at all time points (209.7 ± 17 [control] vs. 95.7 ± 21.4 [day 7], 93.0 ± 16.9 [day 14], and 93.7 ± 12.3 [day 21] nmol/mg protein; *P* < 0.05) (Fig. 4A). These observations were mirrored by a decrease in intracellular neutral lipid (TG) staining with oil red O, after 7 days of DMSO treatment (Fig. 4D).

Removal of fatty acids from the hepatocyte can occur either by secretion of TG from the cell or through oxidation pathways. 3OHB concentrations were significantly increased in HepG2 cell medium after treatment with 1% DMSO for 7 and 14 days (8.4 ± 2.7 [control] vs. 13.4 ± 1.7 [day 7], 12.12 ± 2.4 [day 14] μmol/mg protein; *P* < 0.05) (Fig. 4B) in comparison with untreated control cells. Unlike primary hepatocytes, HepG2 cells secrete very low levels of TG (Wang et al. 1988). While DMSO treatment appeared to increase the TG concentration in the medium from HepG2 cells in at time‐dependent manner, this did not achieve statistical significance (Fig. 4C).

**Figure 5:**
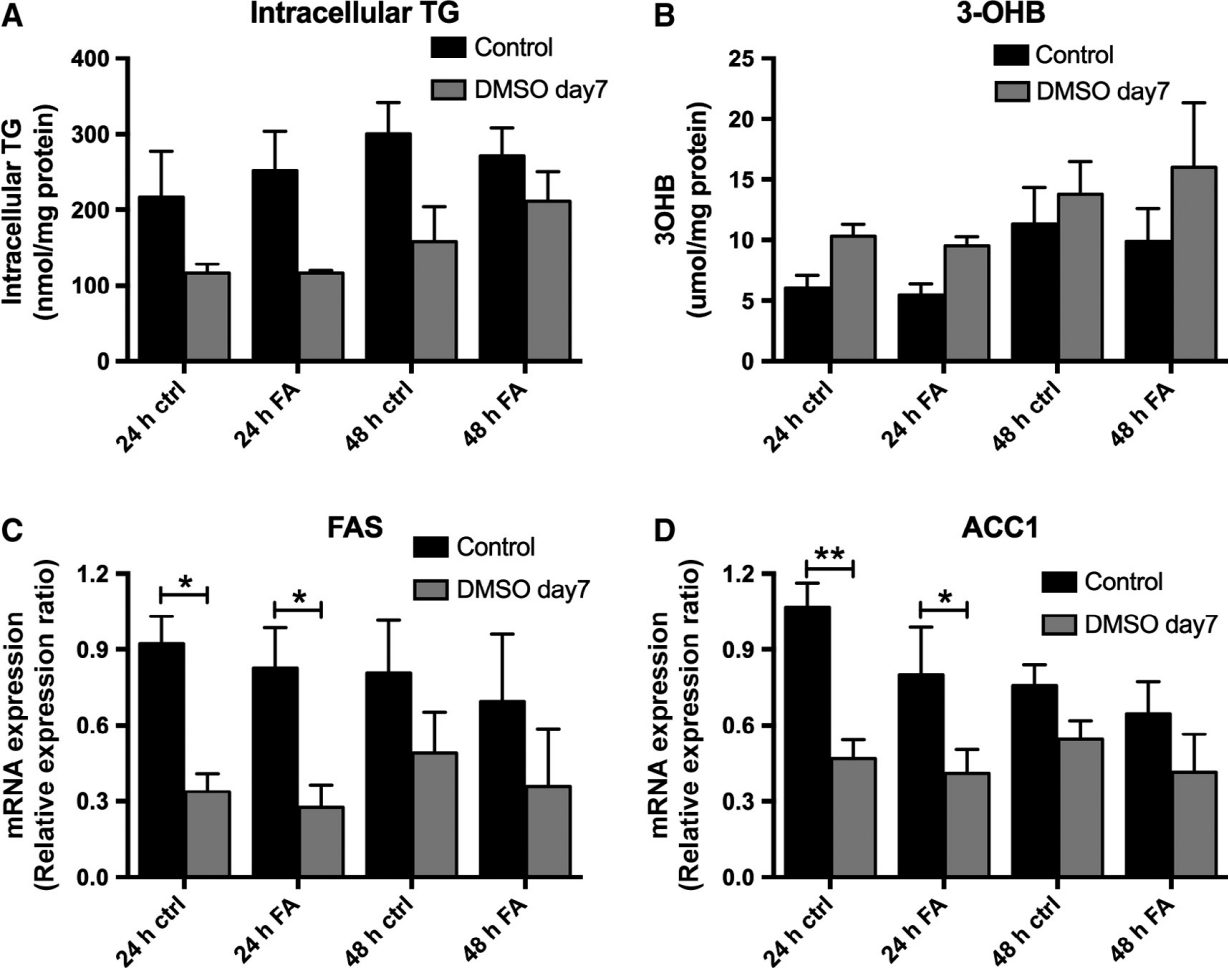
Additional exogenous fatty acid treatment (45 μmol/L oleic acid, 30 μmol/L palmitic acid, and 25 μmol/L linoleic acid), does not significantly change intracellular TG and media 3OHB levels in HepG2 cells after 7 days of DMSO treatment (A and B). Furthermore, exogenous fatty acid treatment has no additional effect in the mRNA expression of FAS and ACC1 after 7 days of DMSO treatment (C and D). Data presented are mean ± SD of *n* = 3 experiments. The relative expression ratio was calculated using the following: Ratio = ΔCt_[target]_/ΔCt_[ref]_ (Pfaffl 2001) and expression values were normalized to 18 sec.

In order to determine whether fatty acid supplementation in addition to DMSO treatment had any further impact upon the lipid metabolic phenotype, control and DMSO‐treated cells (7 days) were treated with 45 μmol/L oleic acid, 30 μmol/L palmitic acid, and 25 μmol/L linoleic acid (OPL; physiological ratio 45%:30%:25%) for 24 or 48 h. We observed no further effect of fatty acid treatment for either 24 or 48 h on all markers of lipid metabolism (Fig. 5A–D).

### DMSO treatment changes glucose metabolism in HepG2 cells

**Figure 6:**
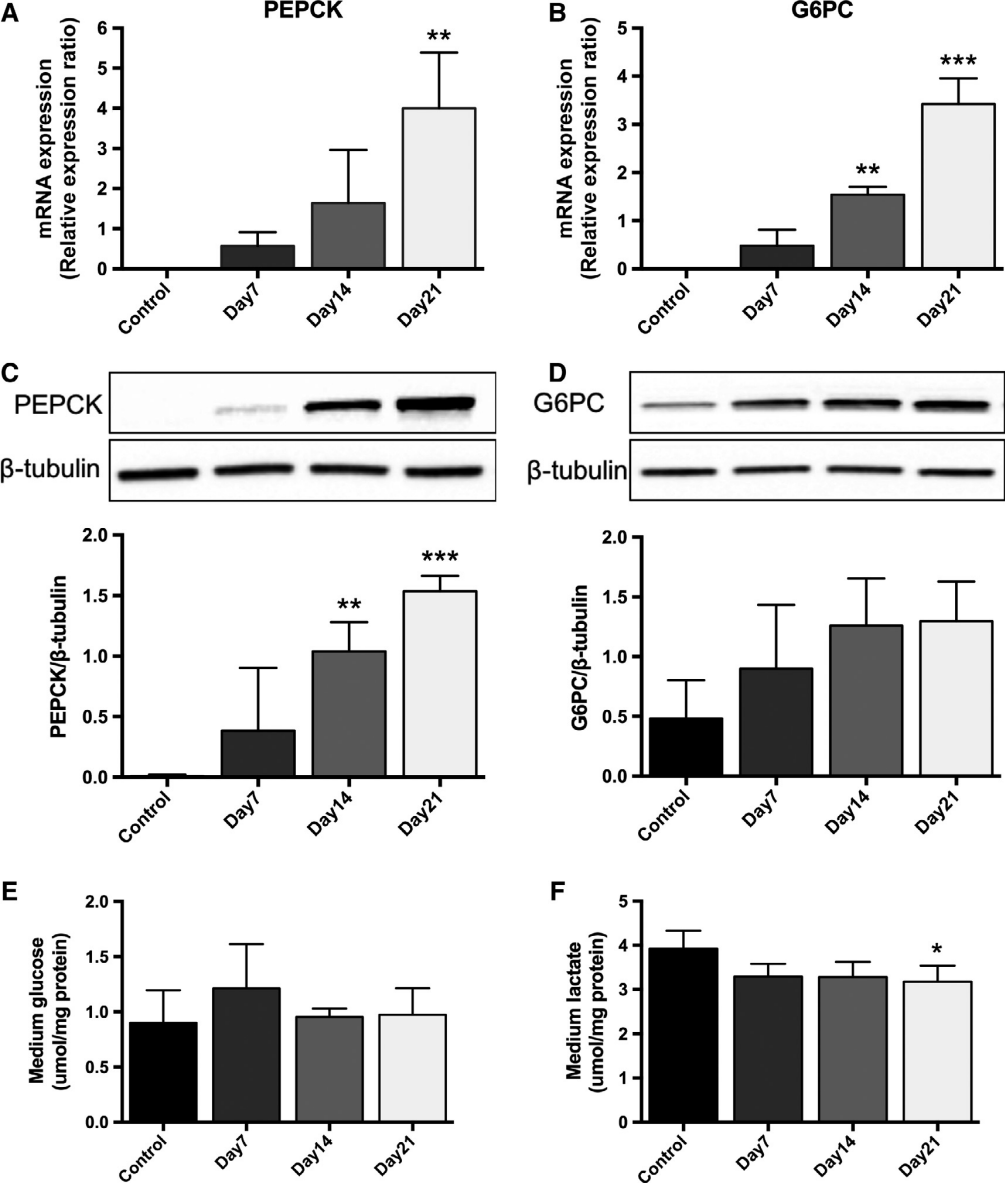
DMSO treatment significantly increases the mRNA expression of phosphoenolpyruvate carboxykinase (PEPCK) and glucose‐6‐phosphatase (G6Pase) in a time‐dependent manner in HepG2 cells (A and B). Changes in mRNA expression were mirrored by changes at the protein level and the protein expression levels of PEPCK and G6Pase were normalized against *β*‐tubulin (C and D). Furthermore, while glucose concentration in the media did not change significantly (E), DMSO treatment decreased media lactate concentrations in HepG2 cells. Data presented are mean ± SD of *n* = 3–5 experiments, *P < 0.05, **P < 0.01, ***P < 0.001, control versus day of DMSO treatment. The relative expression ratio was calculated using the following: Ratio = ΔCt_[target]_/ΔCt_[ref]_ (Pfaffl 2001) and expression values were normalized to 18 sec.

In untreated control HepG2 cells, the mRNA expression of genes driving glucose production, phosphoenolpyruvate carboxykinase (PEPCK), and glucose‐6‐phosphatase (G6Pase) was low (Fig. 6A and B). DMSO treatment significantly increased the mRNA expression of both genes in a time‐dependent manner (PEPCK: 0.003 ± 0.0008 [control] vs. 0.56 ± 0.35 [day 7], 1.64 ± 1.32 [day 14], 4.0 ± 1.38 [day 21], *P* < 0.05; G6Pase: 0.004 ± 0.0009 [control] vs. 0.48 ± 0.33 [day 7], 1.54 ± 0.16 [day 14], 3.42 ± 0.53 [day 21], *P* < 0.05, data expressed as relative expression ratio). Changes in mRNA expression were mirrored by changes at the protein level (Fig. 6C and D). While glucose concentration in the media did not change significantly (Fig. 6E), DMSO treatment (21 days) decreased media lactate concentrations (3.93 ± 0.4 [control] vs. 3.18 ± 0.36 [day 21]* *μmol/mg protein; *P* < 0.05) (Fig. 6F).

## Discussion

Liver diseases are major causes of mortality and morbidity in humans (Younossi et al. 2013), therefore in order for effective treatment strategies to be developed, the cellular mechanisms involved in their etiology need to be defined. As a consequence, there is a necessity for in vitro cellular models that recapitulate human hepatic metabolism. Primary cultures of human hepatocytes still represent the gold standard, but their limitations including availability, inability to proliferate, and donor variability hamper their utility. In addition, in many circumstances the primary culture tissues that are available are from individuals with underlying liver disease and therefore their physiology and function may already be altered. Alternative models of human hepatocytes, including cell lines that have been derived from hepatomas (e.g., HepG2, Huh7.0, Hep3b), offer the potential to be useful for metabolic studies, but have limitations, principally in understanding how closely they reflect human metabolism. Previous reports have suggested that DMSO treatment induces differentiation and drug metabolism in human hepatoma cell lines (Isom et al. 1985; Choi et al. 2009), although the molecular mechanisms are not fully understood.

**Figure 7:**
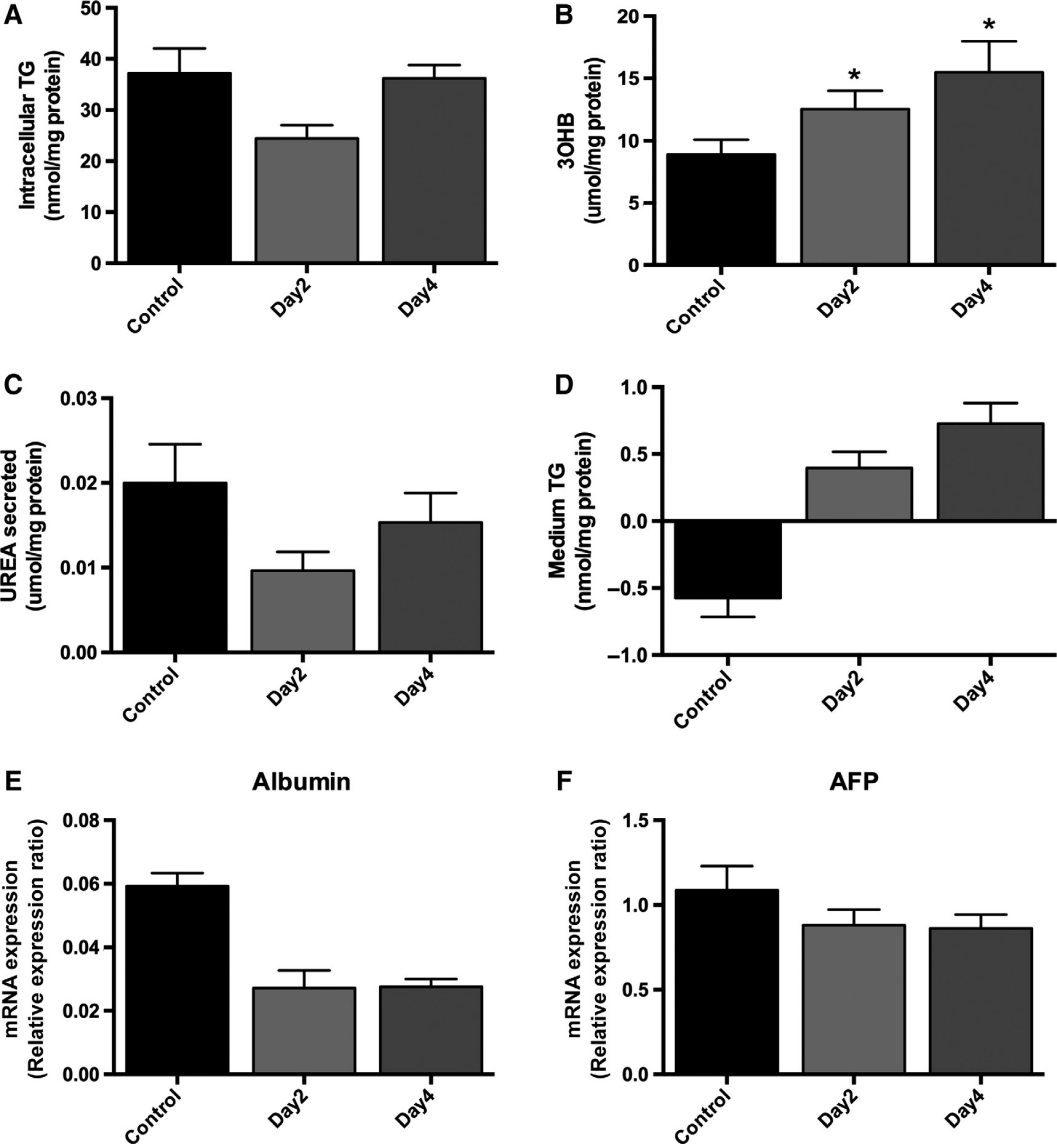
DMSO treatment significantly increases 3OHB levels (B), but it is without effect on intracellular TG content (A), secreted urea (C), or media TG (D) in HepG2 cells after 2 and 4 days of treatment. Furthermore, DMSO treatment does not significantly change the mRNA expression of albumin (E) and AFP (F) after 2 and 4 days of treatment. Data presented are mean ± SD of *n* = 3 experiments, *P < 0.05, control versus day of DMSO treatment. The relative expression ratio was calculated using the following: Ratio = ΔCt_[target]_/ΔCt_[ref]_ (Pfaffl 2001) and expression values were normalized to 18 sec.

By supplementing 1% DMSO to cell culture medium of HepG2 cells, we observed metabolic functionality over a time course of 21 days, with optimal functionality occurring at 7 days. Earlier time points were also tested (2 and 4 days of treatment), but little effect was observed (Fig. 7A–F). In particular, we have shown that DMSO treatment reduces intracellular TG accumulation which appears to be mediated through the suppression of lipogenic gene expression. In contrast, we observed a significant increase in the expression of key genes involved in intracellular glucose metabolism.

Hepatocyte functionality has previously been assessed by the measurement of markers involved in cell differentiation and viability. The most frequently measured markers include albumin, alpha‐1‐antitrypsin, transthyretin, and HNF1A (Sainz and Chisari 2006; Nikoozad et al. 2014). We measured markers of liver functionality that have been shown to be key in human hepatocyte physiology and found robust changes in the expression of these genes that more closely resembled primary human hepatocytes. Although we did not directly assess cell viability, previous reports have demonstrated DMSO treatment does not cause cytotoxicity in concentrations up to 2% (Wang et al. 2012), double the concentration used in our experiments. One of the advantages of using hepatoma cell lines is their ability to proliferate reflecting their origin from hepatocellular carcinoma. Among the reported effects of DMSO is its ability to decrease proliferation and induce or maintain differentiation (Sainz and Chisari 2006). Following DMSO treatment, we observed a reduction in the malignant phenotype as evidenced by a significant decrease in AFP mRNA expression. DMSO is known to upregulate tumor suppressor PTEN expression in leukemic HL60 cells (Lee et al. 2005) and tumor suppressor protein HLJ1 in lung cancer cell lines (Wang et al. 2012). Taken together, treatment with 1% DMSO in cell culture medium has the ability to enhance hepatocyte functionality of hepatoma‐derived cell lines to be more closely resemble human primary hepatocytes.

Lipid synthesis in the context of hepatocellular carcinoma (HCC) has been reported to be associated with increased HCC survival allowing it to survive at times of metabolic stress (Wang et al. 2016). The decrease in lipid synthesis that we observed provides further evidence that we have reduced the malignant phenotype of the HepG2 cells. Furthermore, rat primary hepatocytes have been reported to have lower levels of intracellular lipid content compared to HepG2 cells. In our study, we have clearly demonstrated that DMSO treatment affects lipid metabolism. The mRNA expression of genes involved in de novo lipogenesis (FAS, ACC1, ACC2) and TG synthesis (DGAT1, DGAT2) was decreased with a consequent reduction in intracellular TG accumulation. Song et al. (2012) demonstrated that low‐dose DMSO (0.01%) treatment of HepG2 cells decreased palmitate‐induced TG accumulation; however, in the absence of palmitate supplementation and in contrast to our findings, intracellular TG content did not differ between untreated and DMSO‐treated cells. It is possible that the difference between these data and our observations are a reflection of the different doses of DMSO used in the respective protocols.

Fatty acid oxidation is impaired in HepG2 cells compared to parenchymal hepatocytes, as measured by carnitine palmitoyl‐transferase activity (Agius 1988). Consistent with this, Gibbons et al. (1994) failed to detect any ketone body formation in olate‐treated HepG2 cells. In our study, fatty acid oxidation in DMSO‐treated HepG2 cells was increased compared to untreated cells, even in the absence of exogenous fatty acids and this may have contributed to the observed decrease in intracellular TG accumulation.

Previous studies have reported TG secretion from HepG2 cells to be impaired when compared to primary rat hepatocytes (Wang et al. 1988), although culture conditions may also have an effect (e.g. glucose concentrations, variability in fetal serum) (Clavey et al. 1999). In our study, we demonstrated that DMSO‐treated cells had increased TG levels in the cell medium compared to untreated controls, in a time‐dependent manner, which again suggests the adoption of a more primary hepatocyte‐like phenotype.

The liver plays an important role in glucose metabolism regulating the balance between exogenous glucose utilization and endogenous production through gluconeogenesis. Indicative of their HCC origin, HepG2 cells have impaired glucose production, as demonstrated by downregulated gluconeogenic gene expression (Wang et al. 2013). In our study, DMSO treatment induced gluconeogenesis in a time‐dependent manner, as indicated by increased PEPCK and G6Pase expression. Carbohydrate metabolism differs in hepatoma cells compared to primary hepatocytes as the former maintain energy production through anaerobic glycolysis (Hugo‐Wissemann et al. 1991). We observed a significant decrease in lactate production after 7 days of DMSO treatment, again suggestive of a more hepatocyte‐like phenotype.

While we have demonstrated robust findings, there are some limitations to our experiments. They were undertaken using standard culture medium containing 25 mmol/L glucose (4.5 g/L). These levels of glucose are nonphysiological and indeed may induce cellular stress and inflammation (Green et al. 2015). This needs to be considered when interpreting our data, although all relevant control cells (and primary hepatocytes) were cultured in the same glucose concentrations. We did not directly assess cell proliferation directly, although others have reported that DMSO at concentrations up to 0.1% enhances proliferation in HepG2 cells (Song et al. 2012); however, at concentrations exceeding 0.5% proliferation rates decrease, though viability is not impaired at concentrations below 2% (Wang et al. 2012). While we have robustly demonstrated the impact of DMSO treatment, cellular confluence is a natural phenomenon and untreated (control) HepG2 cells continue to proliferate rapidly, resulting in aberrant monolayer and increased cell death in the postconfluent stage, as measured by extensive floating cells in the cell medium (data not shown). As a consequence, it was not possible to match experimental treatment times with non‐DMSO‐treated control cells.

In conclusion, we have shown that 1% DMSO treatment attenuates lipid accumulation, induces fatty acid oxidation and TG secretion, and augments gluconeogenesis in HepG2 cells such that they more closely resemble primary human hepatocytes. This represents a signficant development and enhancement of the currently available in vitro model systems to study hepatocy biology and has important implications, not only for the study of the pathological processes that contribute to disease, but also their response to specific therapeutic interventions.

## Conflict of Interest

We confirm that this manuscript has not been presented or published elsewhere. N. N., C. J. P., P. J. G., L. H., and J. W. T. do not have any conflict of interest to declare.
